# A Waterborne Outbreak of *Escherichia coli* O157:H7 Infections and Hemolytic Uremic Syndrome: Implications for Rural Water Systems^1^

**DOI:** 10.3201/eid0804.000218

**Published:** 2002-04

**Authors:** Sonja J. Olsen, Gayle Miller, Thomas Breuer, Malinda Kennedy, Charles Higgins, Jim Walford, Gary McKee, Kim Fox, William Bibb, Paul Mead

**Affiliations:** * Centers for Disease Control and Prevention, Atlanta, Georgia, USA; † Wyoming Department of Health, Cheyenne, Wyoming, USA; ‡ Wyoming Department of Agriculture, Cheyenne, Wyoming, USA; # United States Environmental Protection Agency, Cincinnati, Ohio, USA

**Keywords:** Escherichia coli O157:H7, waterborne transmission, epidemiology, drinking water, antibody

## Abstract

In the summer of 1998, a large outbreak of *Escherichia coli* O157:H7 infections occurred in Alpine, Wyoming. We identified 157 ill persons; stool from 71 (45%) yielded *E. coli* O157:H7. In two cohort studies, illness was significantly associated with drinking municipal water (town residents: adjusted odds ratio=10.1, 95% confidence intervals [CI]=1.8-56.4; visitors attending family reunion: relative risk=9.0, 95% CI=1.3-63.3). The unchlorinated water supply had microbiologic evidence of fecal organisms and the potential for chronic contamination with surface water. Among persons exposed to water, the attack rate was significantly lower in town residents than in visitors (23% vs. 50%, p<0.01) and decreased with increasing age. The lower attack rate among exposed residents, especially adults, is consistent with the acquisition of partial immunity following long-term exposure. Serologic data, although limited, may support this finding. Contamination of small, unprotected water systems may be an increasing public health risk.

*Escherichia coli* O157:H7 is now a well-recognized cause of human illness. Although outbreaks of *E. coli* O157:H7 infections are frequently associated with food or milk derived from cattle, other sources, including fresh fruits and vegetables and water, have been implicated (*1*). In the United States, the first reported drinking water outbreak of *E. coli* O157:H7 infections occurred in 1989 in rural Missouri (*2*). Since this outbreak, six others have been associated with drinking water. Three were small and occurred in a camp, a recreational vehicle park, and a well (Centers for Disease Control and Prevention [CDC], unpub. data). More recently, three highly publicized drinking water outbreaks of *E. coli* O157:H7 infections (one each in Wyoming, New York, and Canada), have focused increased attention on the safety of drinking water (*3*,*4*). Here we summarize the results of the outbreak investigation in Wyoming.

During late June 1998, physicians near Alpine, Wyoming, noted an increase in bloody diarrhea among town residents. Alpine is a small town (pop. <500) on the Wyoming-Idaho border that is frequented by tourists to Grand Teton and Yellowstone National Parks. By July 9, *E. coli* O157:H7 had been isolated from stool samples from 14 persons, including residents of Wyoming, Utah, and Washington. On July 11, CDC and Wyoming health officials began an investigation to determine the magnitude of the outbreak and the source of *E. coli* O157:H7 infections.

## Methods

### Case Finding and Hypothesis Generation

To identify patients, Wyoming health officials contacted area physicians and health officials in neighboring states. Interviews identified ill persons in Alpine, as well as ill persons from outside Wyoming who had attended a large family reunion in Alpine June 26-28. Patients were routinely interviewed about a wide variety of potential exposures, and stool samples were collected for laboratory confirmation. Environmental health inspectors in Wyoming conducted an assessment of retail food distribution in Alpine. This information was used to develop questionnaires for the subsequent cohort studies.

### Family Reunion Cohort Study

On July 11 and 12, we conducted a cohort study of persons from out-of-state families who had attended the family reunion in Alpine from June 26 to 29. Using a questionnaire administered by telephone, we asked reunion attendees about illness and exposure to certain foods and municipal water during their stay in Alpine. All family members were questioned individually except children too young to be interviewed; parents responded for these children. A case was defined as diarrhea (>3 loose stools in a 24-hour period) with onset after June 26 in a person attending the family reunion.

### Alpine Cohort Study

To confirm the results of the smaller family reunion cohort and to estimate the attack rate in the community, we conducted a cohort study of all residents of Alpine during July 13-16 . Using a questionnaire administered by telephone, we asked residents about recent gastrointestinal symptoms and exposure to various meats and Alpine municipal water during the 7-day period June 25 to July 1. We collected information on consumption of tap water or beverages made with tap water, the average number of glasses drunk in the week, and water filtration practices. Telephone numbers were called at least twice (once during the day and once at night) before being deemed a “no answer.” A case was defined as a) a stool culture yielding *E. coli* O157:H7, or b) diarrhea (>3 stools in a 24-hour period) with onset after June 25 in an Alpine resident who was in town between June 25 and July 1.

### Laboratory Investigation

All stool samples were sent on ice to the Wyoming state laboratory, where they were plated on sorbitol-MacConkey agar (*5*). Samples producing sorbitol-negative colonies were selected and tested for O157 antigen, and positive samples were then assayed for H7 antigen by latex agglutination (*6*). Specimens identified as *E. coli* O157:H7 were sent to the Utah Department of Health State Laboratory for subtyping by pulsed-field gel electrophoresis (PFGE) (*7*). Additional stool samples were collected serially from day-care attendees, food handlers, and health-care workers whose first stool culture yielded *E. coli* O157:H7; subsequent stool samples were tested for Shiga toxin by using only the Premier EHEC assay (Meridian Diagnostics Inc., Cincinnati, OH) (*8*). Serum was collected from town residents to test for immunoglobulin (Ig) G and IgM antibodies to O157 lipopolysaccharide by enzyme-linked immunosorbent assay (ELISA) at CDC (*9*). An antibody >1:320 for IgM or >1:160 for IgG was considered positive.

### Environmental Investigation

Representatives from the Wyoming State Department of Agriculture and the U. S. Environmental Protection Agency (EPA) inspected the Alpine municipal water system and reviewed testing and safety measures. On July 14, 2001, water samples were collected from the outlet of the storage tank and several points in the distribution line. Deer and elk fecal samples were collected in the area above the spring collection system. Water samples were sent overnight to the Cincinnati EPA laboratory and analyzed for bacterial contamination within 24 hours of collection. Water samples were tested for total and fecal coliforms and *E. coli* by using m-ENDO Medium (*10*). Animal fecal samples were tested for *E. coli* O157:H7 at the Wyoming state laboratory as described above for human samples.

### Statistical Analysis

In the univariate analysis, relative risks (RR) and 95% confidence intervals (CI) were computed by using Epi Info (version 6.04, CDC, Stone Mountain, GA). Variables found to be significantly associated with disease at the univariate level were examined in a logistic regression model (LogXact version 2, Cytel Software Corporation, Cambridge, MA). The minimum duration of fecal shedding was estimated as the time between the onset of symptoms to the last positive Shiga toxin result before two negative results. Data for children and adults were examined by using Kaplan-Meier survival analysis; curves were compared by using the log-rank test (SAS version 6.12, SAS Institute Inc., Cary, NY).

## Results

### Case Finding

**Figure 1:**
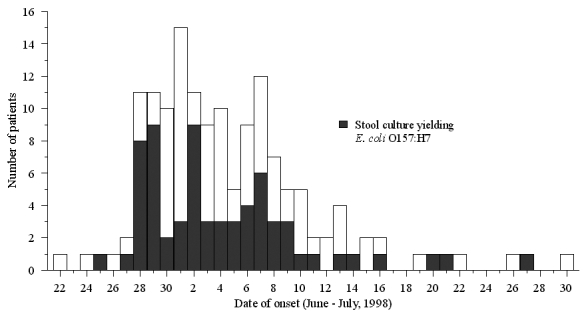
Cases of diarrhea by date of onset and *Escherichia coli* O157:H7 culture status, Alpine, Wyoming, June to July 1998.

We identified 157 ill persons from 15 states. The dates of illness onset ranged from June 25 to July 27 for patients with culture-confirmed infection and June 22 to July 30 for patients with culture-negative stools (Figure 1). Hemolytic uremic syndrome developed in four persons: a 15-month-old girl, a 17-month-old boy, a 4-year-old boy, and a 36-year-old woman. All have since recovered. Fifty-seven isolates were subtyped by PFGE; the patterns were indistinguishable.

Review of initial interviews showed that ill persons reported only infrequent exposure to some food and drink items known to be associated with *E. coli* O157:H7 transmission, including raw milk and apple cider. Wyoming state environmental health inspectors determined that no recently recalled food products, including ground beef, had been shipped to stores in Wyoming. This information was verified by U.S. Food and Drug Administration and U.S. Department of Agriculture officials.

### Family Reunion Cohort

Twelve families consisting of 44 persons attended the family reunion in Alpine. Of the 41 (93%) persons interviewed, 12 met the case definition; one patient's stool sample yielded *E. coli* O157:H7. Initial interviews showed that all food and drink items, except water, were purchased out of state.

The overall attack rate was 29%: 33% among males, 24% among females, and 50% among children <5 years old. The only exposure significantly associated with illness was drinking Alpine municipal water. Persons who drank Alpine municipal water were nine times more likely to become ill than were persons who did not drink the water (95% CI=1.3-63.3). The attack rate among reunion members who drank Alpine municipal water during the weekend was 50%. Attack rates for persons who drank Alpine municipal water did not vary by age (*14*) (Table 1)

**Table 1:** Attack rates among persons exposed to municipal water, by age group, Alpine, Wyoming, June–July 1998

Age group (years)	Resident cohort N (%)	Family reunion cohort (nonresidents) N (%)
All ages	48/207 (23)	11/22 (50)
<9	10/30 (33)	3/6 (50)
10-19	7/26 (27)	2/5 (40)
20-39	11/55 (20)	3/5 (60)
40-59	16/63 (25)	3/6 (50)
>60	4/33 (12)	0/0

### Alpine Cohort

The 1997-98 Alpine telephone directory listed 490 phone numbers with the Alpine prefix; of the 287 household numbers, we reached 146 (51%). Twelve of the households reached were excluded because the family had been out of town during the exposure period, June 25 to July 1. A total of 319 persons were living in the remaining 134 households during the exposure period; 284 (89%) were available for interview. Of the 284 respondents, 139 (49%) were male, and the median age was 39 years (range <1 to 92). For 192 (68%) interviewees, Alpine municipal water was their source of tap water at home.

Fifty-four (19%) persons met the case definition; 18 (33%) had stool samples yielding *E. coli* O157:H7. The attack rate was almost two times higher in women (25%) than in men (13%); however, in the week before illness, women were no more likely than men to drink any or more water.

In univariate analysis, 5 of 16 exposure variables were significantly associated with illness (except when noted, all exposures refer to the period June 25 through July 1): playing in a sprinkler, playing with water guns, being serviced by municipal water at home, drinking municipal water, and drinking municipal water during June 26 to 28 (Table 2). Since drinking Alpine municipal water was a broader exposure than, and not as strong a risk factor for illness as, drinking municipal water during June 26 to 28, it was excluded in multivariate analysis. In the multivariate model, the only variable that remained significantly associated with illness was drinking municipal water on the weekend of June 26 to 28 (Table 3; OR 10.1; 95% CI=1.8-56.4).

**Table 2:** Univariate analysis of exposures in Alpine resident cohort study, Alpine, Wyoming, June–July 1998a

Selected exposures^a^	Proportion ill		Relative risk (95% CI)
	Exposed	Nonexposed	
Played in sprinkler	10/29	44/254	2.0 (1.1 - 3.5)
Played with water guns	7/19	47/264	2.1 (1.1 - 3.9)
Serviced by municipal water at home	45/192	9/92	2.4 (1.2 - 4.7)
Drank municipal water^a^	8/211	3/68	5.2 (1.7 - 16.0)
Drank municipal water June 26-28^b^	8/181	2/62	8.2 (2.1 - 32.8)
Venison consumption	3/13	51/271	1.2 (0.4 - 3.4)
Elk consumption	10/44	44/239	1.2 (0.7 - 2.3)
Jerky consumption	4/25	49/256	0.8 (0.3 - 2.1)
Hamburger consumption	29/147	21/111	1.0 (0.6- 1.7)
Pink hamburger consumption	0/7	27/130	undefined, p=0.2

^a^Except when noted, all exposures refer to the period June 25 through July 1, 1998. ^b^Only 8 (4%) of 211 persons who drank municipal water reported boiling it. CI = confidence intervals.

**Table 3:** Multivariate analysis of exposures in Alpine resident cohort study, Alpine, Wyoming, June–July 1998

Exposure	Odds ratio (95% CI)
Played in sprinkler	1.5 (0.6-3.9)
Played with water guns	1.4 (0.5-4.3)
Serviced by municipal water at home	1.2 (0.4-3.8)
Drank municipal water June 26-28	10.1 (1.8-56.4)

CI = confidence intervals.

Overall, the attack rates among residents who drank Alpine municipal water were 23% during June 25 to July 1 and 27% during the 3-day period of June 26-28. Attack rates among females or males for persons who drank Alpine municipal water did not differ significantly. However, although not significant, the risk for illness decreased with increasing age (Table 1). Among persons who drank municipal water, the average number of glasses of water and the risk for illness were not statistically associated (Kruskal-Wallis test, p=0.7).

### Laboratory Investigation

Twenty persons with culture-confirmed infection were followed with serial stool cultures to examine the duration of fecal shedding; 14 were children <10 years old in day care and 6 were adults aged 16 to 60 years (including 4 adult food handlers and 1 health-care worker). The median length of fecal shedding was 9 days among children (range 1 to 35) and 7.5 days among adults (range 2 to 36), a difference that was not statistically significant (p=0.63). Among children <5 years old, the median length of fecal shedding was 9 days (range 1 to 35).

**Figure 2:**
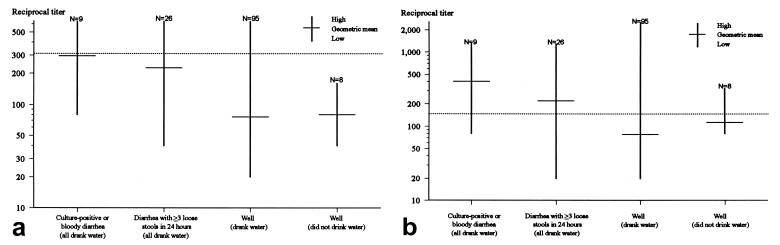
Reciprocal geometric mean and range of (a) immunoglobulin (Ig)M and (b) IgG (b) antibody titers to O157 lipopolysaccharide. The dotted line indicates the positive cutoff (IgM >1:320 and IgG >1:160).

Serum samples were collected from 129 town residents 16 to 27 days after the most likely time of exposure, July 27. Of the 129 persons, 57 (44%) were male, and the mean age was 42 years (range 3 to 78 years). Twenty-six (20%) persons had illness that met the Alpine cohort case definition (all drank Alpine municipal water), and nine of these were either culture positive or had bloody diarrhea. Of the other 103 (80%) well persons, 95 drank municipal water and 8 did not. IgM and IgG geometric mean titers were highest in persons with culture- positive *E. coli* O157:H7 or bloody diarrhea, followed by persons with diarrhea, and then well persons (Figure 2). The geometric mean IgM titer was <1:320 and the IgG titer >1:160 for ill persons. Three of eight well persons who did not drink Alpine municipal water during the outbreak had IgG titers >1:160.

### Environmental Investigation

The Alpine municipal water system was supplied by an underground spring. A series of small boxes connected by perforated pipes 7 to 10 feet below ground level collected water from an unconfined aquifer and routed it to an underground concrete storage tank. Pipes feeding off this tank then delivered unchlorinated water to the town. Sanitary surveys conducted in 1992 and 1997 indicated a potential risk of contamination from wildlife as well as surface water. Although Alpine was in compliance with the Total Coliform Rule, which requires one safe total coliform result each month, there were several positive readings in April 1998 (1/5 positive), May 1998 (4/7 positive), and June 1998 (2/10) just before the outbreak. On inspection after the outbreak, the spring was found to be under the influence of surface water; a large pool of water was found in the area over the water collection pipes, probably the result of a late snow melt combined with heavy rains and ground water outfalls. Numerous deer and elk feces were present in the area; the animals came to the pool to drink. *E. coli* O157:H7 was not isolated from any of the five deer or elk fecal samples taken on July 14. Water taken from the storage tank on July 14 yielded a total coliform count of 108 CFU/100 mL. *Enterococcus faecium* was isolated from the same water sample, indicating fecal contamination. *E. coli* O157:H7 was not isolated.

## Discussion

In this investigation, we identified more than 150 cases of acute gastrointestinal illness among residents of Alpine and visitors from 14 other states. *E. coli* O157:H7 was confirmed by culture in 71 cases. Four persons, including three children, were hospitalized with hemolytic uremic syndrome; there were no deaths. Illness was significantly associated with drinking unchlorinated water from the Alpine municipal water system in each of two cohort studies.

We believe that the unchlorinated municipal water supply became contaminated when surface water, containing deer and elk feces, leached into the town's unconfined aquifer. Deer and other ruminants can harbor *E. coli* O157:H7 and shed the organism in their feces (*11*,*12*). Although EPA did not detect *E. coli* O157:H7 in water from the storage tank several weeks after the outbreak, the analysis did reveal high coliform counts and the presence of *Enterococcus faecium*. *Escherichia coli* O157:H7 has been shown to survive for prolonged periods in water, especially in cold water, by transforming into a viable but nonculturable state (*13*). In this state the pathogen cannot be isolated by traditional plating methods and therefore may not be detected.

Our serologic findings demonstrate the cross-sectional antibody profile of a population during an outbreak. IgG antibody titers to O157 lipopolysaccharide generally develop within the first week after illness onset and remain elevated for at least 2 months (*9*), whereas IgM antibody titers appear to increase more rapidly and decline within 8 weeks after illness onset (*14*). Consistent with a previous study (*9*), ill persons in our study demonstrated a markedly increased IgG antibody response but not IgM. Although this may reflect the fact that blood was drawn 2 to 4 weeks after exposure, when IgM antibodies may already be decreasing, it more likely reflects the lower specificity of this assay (*9*). While antibody titers to O157 lipopolysaccharide generally correlate with severity of disease (*9*), the titers of persons who do not develop clinical illness are thought to be low. Our findings suggest that among well persons, those exposed may have elevated titers compared with those not exposed.

Several lines of evidence suggest that Alpine residents may have been previously exposed to *E. coli* O157:H7 and as a result may have acquired a degree of immunity to symptomatic infection. Although the geometric mean titer of IgG antibodies was not above 1:160, three (38%) of eight well residents who did not drink municipal water during the current outbreak had titers >1:160. In contrast, studies conducted to establish the specificity of the serologic assay have shown that only 3% to 4% of well persons in the United States have IgG antibodies titers >1:160 (CDC, unpub. data), suggesting a higher than expected baseline rate of seropositivity among Alpine residents. In addition, among persons who were exposed to municipal water on the weekend of June 26 to 28, the attack rate was significantly lower for town residents than for nonresident visitors (Table 1; 27% vs. 50%, p<0.01). Further, although not statistically significant, the attack rate among town residents who drank municipal water decreased with increasing age, whereas it was the same at all age groups among visitors, a finding consistent with older residents' having more opportunity for previous exposure to *E. coli* O157:H7. Although these findings suggest previous exposure to *E. coli* O157:H7, these data were based on small sample sizes. The mechanism of previous exposure may have been through episodic contamination of the water supply or eating wild game; 20% of persons interviewed reported having eaten elk, venison, or jerky during the week in question.

The median duration of shedding of *E. coli* O157:H7 by persons in this outbreak was slightly shorter than previously reported (*15*–*18*). The difference may be attributable to different methods used to estimate the endpoint of shedding; because stool samples were not collected at regular intervals in our study, we chose a conservative estimate of this endpoint, i.e., the last positive result.

We identified several methodologic limitations in this investigation. The first was nonresponse bias since only half the telephone numbers listed in Alpine were reached. However, since Alpine is a seasonal community with many part-year residents and rental properties, it is likely that we reached most of the households with members present at the time of the outbreak. An additional concern was the potential for household clustering in our town cohort study since several members within a household were interviewed. However, this effect was examined by using generalized estimating equation models and showed no appreciable difference from the simpler logistic regression models.

This outbreak highlights the importance of *E. coli* O157:H7 as a waterborne pathogen. The organism has a low infectious dose (*19*), which allows water to act as an efficient vector, and watersheds that are vulnerable to infiltration by animals run the risk of contamination. Of 18 waterborne outbreaks of *E. coli* O157:H7 infections reported to CDC from 1982 to 1998, five were caused by contaminated drinking water (CDC, unpub. data). All five of these outbreaks involved small water systems or wells that supplied rural townships or camps. More recently, in September 1999, a large waterborne outbreak of *E. coli* O157:H7 infections occurred at a county fair in New York (*3*). In that outbreak, the drinking water was likely contaminated when cow manure seeped into a shallow, unchlorinated well after a large rainstorm. Because of underreporting and underdiagnosis, reported outbreaks probably represent a small fraction of the true number of *E. coli* O157:H7 outbreaks associated with drinking water in the United States. In addition to data from domestic outbreaks, contaminated water has been implicated as the cause of at least four outbreaks in other countries (*4*,*20*–*22*), including a massive outbreak with over 1,400 illnesses in Canada in May 2000 (*4*), and as a risk factor for sporadic infection (*23*,*24*). These illnesses could have been prevented by properly protecting the water sources and adequate chlorination.

Small water systems, defined as those that serve fewer than 3,300 people, collectively serve approximately 40 million people, or 15% of the United States population (*25*). Small drinking water systems may be less likely to be adequately chlorinated and to routinely monitor for contaminants (*25*). The outbreak reported here confirms the potential of these small, unprotected and unchlorinated water systems to be an important source of infection with *E. coli* O157:H7 and other pathogens. Stronger enforcement of existing regulations and perhaps broadening of current regulations, such as the proposed ground water rule designed to prevent illness from drinking water from ground water sources through disinfection (*26*), are needed to protect rural drinking water systems in the United States.
